# Prognostic and diagnostic significance of copeptin in acute exacerbation of chronic obstructive pulmonary disease and acute heart failure: data from the ACE 2 study

**DOI:** 10.1186/s12931-017-0665-z

**Published:** 2017-11-03

**Authors:** Jacob A. Winther, Jon Brynildsen, Arne Didrik Høiseth, Heidi Strand, Ivar Følling, Geir Christensen, Ståle Nygård, Helge Røsjø, Torbjørn Omland

**Affiliations:** 1 Division of Medicine, Akershus University Hospital, Lørenskog, Norway; 2 Institute of Clinical Medicine, University of Oslo, Oslo, Norway; 3 Institute for Experimental Medical Research, Oslo University Hospital and University of Oslo, Oslo, Norway; 4 Division of Diagnostics and Technology, Akershus University Hospital, Lørenskog, Norway; 5 Bioinformatics Core Facility, Institute for Medical Informatics, Oslo University Hospital and University of Oslo, Oslo, Norway

**Keywords:** Copeptin, NT-proBNP, COPD, Heart failure, Epidemiology, Dyspnea, Hyponatremia, Vasopressin, Antidiuretic hormone

## Abstract

**Background:**

Copeptin is a novel biomarker that predicts mortality in lower respiratory tract infections and heart failure (HF), but the diagnostic value of copeptin in acute dyspnea and the prognostic significance of copeptin in acute exacerbation of chronic obstructive pulmonary disease (AECOPD) is not clear.

**Method:**

We determined copeptin and NT-proBNP concentrations at hospital admission in 314 patients with acute dyspnea who were categorized by diagnosis. Survival was registered after a median follow-up of 816 days, and the prognostic and diagnostic properties of copeptin and NT-proBNP were analyzed in acute HF (*n* = 143) and AECOPD (*n* = 84) separately.

**Results:**

The median concentration of copeptin at admission was lower in AECOPD compared to acute HF (8.8 [5.2–19.7] vs. 22.2 [10.2–47.9]) pmol/L, *p* < 0.001), but NT-proBNP discriminated acute HF from non-HF related dyspnea more accurately than copeptin (ROC-AUC 0.85 [0.81–0.89] vs. 0.71 [0.66–0.77], *p* < 0.0001). Adjusted for basic risk factors, increased copeptin concentrations predicted mortality in AECOPD (HR per log (ln) unit 1.72 [95% CI 1.21–2.45], *p* = 0.003) and acute HF (1.61 [1.25–2.09], *p* < 0.001), whereas NT-proBNP concentrations predicted mortality only in acute HF (1.62 [1.27–2.06], *p* < 0.001). On top of a basic model copeptin reclassified a significant proportion of patients into a more accurate risk strata in AECOPD (NRI 0.60 [0.19–1.02], *p* = 0.004) and acute HF (0.39 [0.06–0.71], *p* = 0.020).

**Conclusion:**

Copeptin is a strong prognostic marker in both AECOPD and acute HF, while NT-proBNP concentrations predict mortality only in patients with acute HF. NT-proBNP levels are superior to copeptin levels to diagnose acute HF in patients with acute dyspnea.

**Electronic supplementary material:**

The online version of this article (10.1186/s12931-017-0665-z) contains supplementary material, which is available to authorized users.

## Background

Acute dyspnea is a major symptom of cardiac and pulmonary pathology frequently leading to hospital admission. Systemic biomarkers, such as cardiac troponins and B-type natriuretic peptides (BNPs), are useful tools in the diagnostic work-up and risk stratification of patients with acute coronary heart disease and heart failure (HF) [1–3], but the clinical application of biochemical markers in acute exacerbation of chronic obstructive pulmonary disease (AECOPD) is more uncertain due to lack of validation of potential candidates [4].

Copeptin is a novel biomarker that could prove helpful in the differential diagnosis and risk evaluation of patients with acute dyspnea. The function of copeptin is unknown, but the molecule is derived from the 39-amino acid C-terminal fragment of the arginine-vasopressin (AVP) precursor molecule, pre-pro-vasopressin. Copeptin and AVP are released in equimolar amounts and plasma levels correlate well [5]. Thus, copeptin plasma concentrations are most likely regulated by the same mechanisms that have been established for AVP. Under normal conditions AVP secretion is regulated according to plasma osmolality by osmoreceptors in the hypothalamus, but several strong non-osmotic pathways also exist [6]. In particular, arterial under-filling, as observed during heart failure, stimulates AVP secretion via baroreceptors in the carotid sinus and the aortic arch [7]. Pulmonary disorders, including COPD are also associated with elevated AVP levels, possibly due to impaired gas exchange or activation of baroreceptors [8, 9], but the mechanism is not clear. In addition, AVP secretion is increased as part of a general stress response [10]. While analytical challenges and stability issues have made reliable measurements of circulating AVP difficult to achieve [11], copeptin is easily measured and stable in plasma and serum for at least 7 days in room temperature and over several freeze and thaw cycles [5, 12]. The prognostic value of copeptin has already been studied in several medical conditions. Previous studies have found increased copeptin concentrations to be associated with poor prognosis in sepsis and hemorrhagic shock [13, 14], myocardial infarction [15], and chronic HF [16–18]. In patients with lower respiratory tract infections, copeptin predicted mortality more accurately than C-reactive protein (CRP) and leucocyte count [19]. Among patients admitted to hospital with acute dyspnea of various etiologies, copeptin was found to be a strong prognostic marker with superior accuracy compared to BNP and NT-proBNP [20]. The aim of the present study was to compare the prognostic and diagnostic properties of copeptin and N-terminal pro-hormone of BNP (NT-proBNP) in acute HF and AECOPD.

## Methods

### Akershus cardiac examination (ACE) 2 study

The Akershus Cardiac Examination (ACE) 2 Study was designed to assess the diagnostic and prognostic value of circulating biomarkers in patients admitted with acute dyspnea to Akershus University Hospital, Lørenskog, Norway. The primary aim of the ACE 2 study was to analyze the prognostic properties of secretoneurin, and a minimum sample size of 350 patients was originally calculated by power analysis for this purpose [21]. The method of patient recruitment and data collection has also been described in detail previously [22–24]. Patients over the age of 18 years were eligible for inclusion during the first 24 h of admission if acute dyspnea was the primary cause for hospitalization as evaluated by the emergency department physician. Exclusion criteria were dementia and other causes precluding informed patient consent, disseminated malignant disease, acute myocardial infarction or coronary intervention, major surgery within the last 2 weeks, incomplete study blood sampling, and hemoglobin <10 g/dL. Consecutive patients were enrolled between 8 am and 2 pm Monday to Thursday. Clinical information was obtained from physicians on call, hospital records, and directly from the patients by dedicated study personnel who used standardized questionnaires. Echocardiography and spirometry results were registered from hospital records. The ACE 2 study was approved by the Norwegian Regional Committees for Medical and Health Research Ethics (REC) South East (#5.2008.2832) and conducted in agreement with the Declaration of Helsinki. All participants provided written informed consent prior to study enrolment.

### Adjudication of diagnosis and outcome

The final diagnosis of the index hospitalization was established by two senior physicians working independently of each other, and discordant diagnoses were resolved by consensus. The two members of the adjudication committee had no knowledge of study biomarker levels, but they had access to all medical records, including follow-up data and cardiac biomarker measurements such as NT-proBNP and troponin T that were ordered by the treating physician. Patients were first classified into acute HF and non-HF related dyspnea, and then patients in the non-HF group were evaluated with respect to the AECOPD diagnosis. The acute HF diagnosis was determined by the European Society of Cardiology criteria [25], and the AECOPD diagnosis was based on the criteria defined by the Global initiative for Chronic Obstructive Lung Disease (GOLD) [26]. Discordant diagnoses were resolved by consensus. Survival status was recorded from electronic hospital records, which are synchronized with Statistics Norway, until the end of follow-up November 1st, 2012.

### Laboratory analysis

Standard biochemical work-up and arterial blood gas measurements were collected at admission. Glomerular filtration rate (GFR) was estimated by the Chronic Kidney Disease Epidemiology Collaboration (CKD-EPI) formula. Study blood sampling was performed by venipuncture and uniformly processed throughout the study period. Copeptin, N-terminal pro-B-type natriuretic peptide (NT-proBNP) and high-sensitivity cardiac troponin T (hs-TnT) were measured in samples obtained <24 h after hospital admission by commercially available assays: B-R-A-H-M-S Kryptor Copeptin assay by Thermo Fisher Scientific Inc., Clinical Diagnostics, BRAHMS GmbH, 16,761 Hennigsdorf, Germany; and proBNP II and troponin T hs STAT assays by Roche Diagnostics, Penzberg, Germany. Copeptin and hs-TnT were measured in serum while NT-proBNP was analyzed in plasma samples. The copeptin assay had a detection limit of 0.9 pmol/L, a functional sensitivity (inter-analysis variation <20%) above 2.0 pmol/L, and a normal reference range (2.5–97.5 percentile) of 0.9–14.9 pmol/L for healthy adults.

### Statistics

We report continuous variables as mean (± standard deviation [SD]) or median (quartile [Q] 1–3) depending on variable distribution. Differences between groups were compared by Student’s *t* test or Mann-Whitney *U* tests as appropriate. Binary data were compared by the chi-square test and are presented as absolute numbers and percentages. Positively skewed variables, including biomarkers, were log transformed by the natural logarithm to approach normal distribution and to reduce the effect of outliers in regression analysis. Variables associated with copeptin concentration were explored by Spearman’s rank correlation coefficient (rho) and linear regression analysis, and independent associations were determined by multivariate linear regression using stepwise forward selection of variables. Patient survival stratified by admission copeptin and NT-proBNP quartiles was analyzed by Kaplan-Meier plots and compared by the log-rank test. We identified factors associated with mortality by univariate Cox proportional hazard regression analysis. A basic multivariate Cox model of independent risk factors excluding biomarkers was constructed by stepwise forward selection based on the likelihood ratio criterion. The independent prognostic effect of each biomarker was determined by adjusting for the variables in the basic clinical risk model. The area under receiver operating curves (ROC-AUC) was used to ascertain the diagnostic and prognostic accuracies of biomarkers, while the value of adding biomarkers to the basic clinical risk models was investigated by calculating the category free net reclassification index (NRI). ROC-AUCs are presented with 95% confidence interval (CI) computed by bootstrap using 5000 iterations. We considered *p* < 0.05 to be statistically significant and statistical analyses were performed using SPSS for Windows version 22.0 (SPSS, Armonk, NY), STATA version 14 (Stata Corp LP, TX, USA), and R 3.3.3 (R Foundation for Statistical Computing, Vienna, Austria). NRI was calculated using the R package PredictABEL.

## Results

**Fig. 1:**
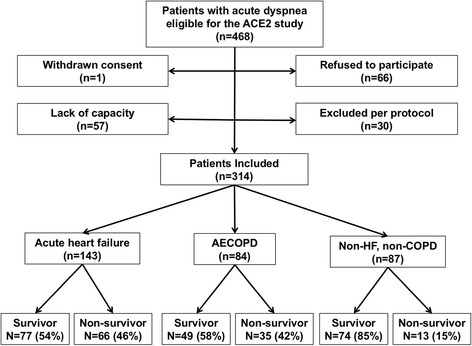
ACE 2 study flow chart

In total, 314 of 468 eligible patients were included in the ACE 2 Study from June 2009 until November 2010. Acute HF was determined to be the primary cause of dyspnea in 143 patients, while 84 patients were diagnosed with AECOPD (Fig. 1). Among patients with dyspnea not related to acute HF or AECOPD (*n* = 87) the most frequent diagnoses were pneumonia (27/87), asthma (16/87), and pulmonary embolism (10/87). Median time from hospitalization to adjudication of diagnosis was 464 days (Q 1–3304–705). The two members of the adjudication committee reached the same diagnosis in 95% (298/314) of the cases, while the remaining 5% (16/324) were resolved by consensus. The baseline characteristics of acute HF and AECOPD patients were consistent with the respective diagnosis (Table 1). Among patients diagnosed with acute HF, chronic HF was previously recognized in 61%, and 43% also had a history of COPD. In the AECOPD group, all patients were previously diagnosed with COPD and the prevalence of chronic HF was 11%.

**Table 1:** Baseline characteristics

	AECOPD (*n* = 84)	Acute HF (*n* = 143)	Non-HF, non-COPD (*n* = 87)	*P* ^*^
Age (years)	69 ± 9	75 ± 11	73 ± 18	<0.001
Male sex	35 (42%)	90 (63%)	39 (45%)	0.002
BMI (kg/m^2^)	24 ± 6	27 ± 6	29 ± 9	0.005
Heart rate (beats/min)	97 ± 18	92 ± 26	91 ± 22	0.107
MAP (mmHg)	102 ± 18	104 ± 21	99 ± 16	0.546
Peripheral edema	31 (37%)	77 (54%)	16 (18%)	0.014
NYHA class IV vs. II-III	47 (56%)	65 (46%)	24 (28%)	0.127
LVEF (%)	60 (50–60)^a^	40 (30–55)	60 (54–60)^a^	<0.001
FEV_1_% of predicted	39 ± 17	n.a^b^	n.a^b^	
FEV_1_/FVC (%)	47 ± 15^a^	n.a ^b^	n.a^b^	
Current smoker	28 (33%)	30 (21%)	27 (31%)	0.039
Diabetes	9 (11%)	43 (30%)	16 (18%)	0.001
Chronic heart failure	9 (11%)	87 (61%)	5 (6%)	<0.001
Coronary artery disease	23 (27%)	78 (55%)	10 (12%)	<0.001
Hypertension	26 (31%)	69 (48%)	25 (29%)	0.011
COPD	84 (100%)	61 (43%)	10 (12%)	<0.001
Beta-blocker	31 (37%)	89 (62%)	19 (22%)	<0.001
ACEi/ARB	27 (32%)	87 (61%)	25 (29%)	<0.001
Diuretic therapy	33 (39%)	104 (73%)	23 (27%)	<0.001
K^+^ (mmol/L)	4.3 ± 0.5	4.4 ± 0.6	4.2 ± 0.5	0.667
Na^+^ (mmol/L)	138 (134–140)	139 (136–141)	138 (136–140)	0.050
eGFR (mL/min)	82 ± 20	61 ± 24	87 ± 31	<0.001
CRP (mg/L)	26 (6–50)	13 (5–35)	16.5 (1–95)	0.019
hs-TnT (ng/L)	18 (9–28)	38 (22–75)	9 (3–23)	<0.001
NT-proBNP (pg/mL)	379 (171–1010)	3600 (1601–8396)	280 (88–1293)	<0.001
Copeptin (pmol/L)	8.8 (5.2–19.7)	22.2 (10.2–47.9)	8.3 (4.3–18.2)	<0.001

Continuous variables are presented as mean ± standard deviation or median (quartile 1–3). Binary variables are presented as absolute numbers and percentages

*Abbreviations*: *ACEi* angiotensin-converting-enzyme inhibitor, *AECOPD* Acute exacerbation of chronic obstructive pulmonary disease, *ARB* angiotensin II receptor blocker, *BMI* Body mass index, *CRP* C-reactive protein, *eGFR* estimated glomerular filtration rate (CKD-EPI), *FEV1* forced expiratory volume in one second, *FVC* forced vital capacity, *HF* heart failure, *hs-TnT* high sensitivity troponin T, *LVEF* left ventricular ejection fraction, *n.a.* not applicable, *NT-proBNP* N-terminal pro-B-type natriuretic peptide, *NYHA* New York Heart Association, *MAP* Mean arterial pressure

^*^ *P* for difference between AECOPD and acute HF

^a^Missing data >10%

^b^Missing data >85%

### Copeptin concentrations and relation to prognosis

**Fig. 2:**
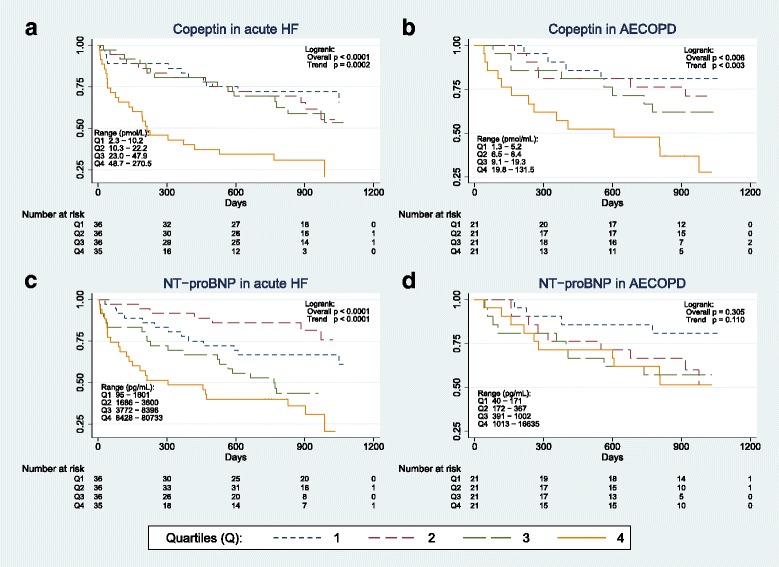
Kaplan-Meier survival plots stratified by biomarker quartiles for (**a**) copeptin in acute HF, (**b**) copeptin in AECOPD, (**c)** NT-proBNP in acute HF, and (**d**) NT-proBNP in AECOPD

**Fig. 3:**
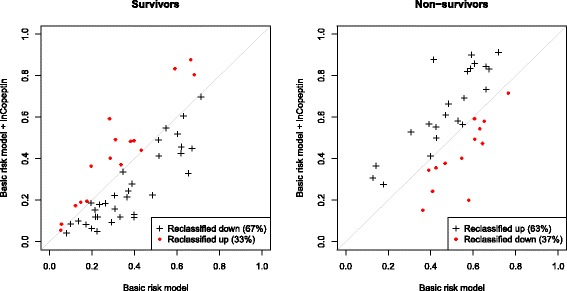
Risk reclassification among AECOPD patients. Estimated risk of death for each patient by the basic model (x-axis) and the enhanced model that also included copeptin (y-axis). “Reclassified up” or “reclassified down” represents an increased or decreased risk of death predicted by the enhanced model as compared to the basic model. The prediction model is improved when survivors are reclassified down, and non-survivors are reclassified up

The prognostic properties of copeptin were analyzed in AECOPD and acute HF separately. After a median follow-up of 2.2 years (813 [356–996] days), 46% (66/143) of HF patients and 42% (35/84) of AECOPD patients had died. According to Kaplan-Meier estimates (Fig. 2) the risk of mortality during follow-up increased among acute HF patients if copeptin or NT-proBNP levels were elevated on hospital admission (*p* < 0.0001 by the log-rank test for both biomarkers). In contrast, only copeptin levels were associated with mortality among patients diagnosed with AEOCPD (Fig. 2; p < 0.0001 by the log-rank test). After adjustment for basic clinical risk factors, as identified by univariate screening (Additional file 1: Table S2), the risk of dying increased by 72% in AECOPD (HR 1.72 [1.21–2.45], *p* = 0.003) and 61% in acute HF (1.61 [1.25–2.09], *p* < 0.001) per log (ln) unit increment of copeptin by multivariate Cox analysis (Table 2). In comparison, one log (ln) unit increase of NT-proBNP increased the risk of mortality by 62% in acute HF (1.62 [1.27–2.06], *p* < 0.001), while no significant predictive effect was found in AECOPD (1.12 [0.88–1.42], *p* = 0.373). Neither copeptin nor NT-proBNP were independently associated with mortality in patients with dyspnea that was not related to CODP or HF (Additional file 1: Table S3). When copeptin and NT-proBNP was included in the same model, the predictive effect of copeptin was significant in patients with AECOPD (HR 1.79 [1.20–2.66], *p* = 0.004), but not in patients with acute HF (1.30 [0.96–1.76], *p* = 0.091). When copeptin was added to the basic clinical risk model, the category free net reclassification index (NRI) was positive in AECOPD (NRI 0.60 [0.19–1.02], *p* = 0.004) and acute HF (0.39 [0.06–0.71], *p* = 0.020). In the AECOPD group, the predicted risk of mortality decreased in 67% of survivors and increased in 63% of non-survivors with the model that included copeptin (Fig. 3). By ROC-AUC analysis, we could not find any statistical difference between the prognostic accuracy of copeptin and NT-proBNP in AECOPD (ROC-AUC 0.67 [0.55–0.79] vs. 0.56 [0.44–0.69], *p* = 0.111) or acute HF (0.66 [0.57–0.75] vs. 0.67 [0.58–0.76], *p* = 0.695). Adding NT-proBNP to the Cox regression model that already included copeptin or vice versa did not significantly alter the overall predictive accuracy of the model, as determined by ROC-AUC or category free NRI, in acute HF.

**Table 2:** Multivariate Cox proportional regression analysis for long-term mortality

	Acute exacerbation of COPD (*n* = 84)		Acute HF (*n* = 143)	
	HR (95% CI)	*P*	HR (95% CI)	*P*
Basic risk factors				
Age (per year)	n.s.		1.04 (1.01–1.07)	0.009
BMI (per kg/m^2^)	0.90 (0.84–0.96)	0.002	0.94 (0.89–1.01)	0.032
Mean arterial pressure (per 5 mmHg)	n.s.		0.99 (0.97–1.00)	0.025
Diabetes mellitus (yes vs. no)	n.s.		2.56 (1.50–4.36)	0.001
COPD (yes vs. no)	n.s.		1.75 (1.04–2.92)	0.035
K^+^ (per mmol/l)	n.s.		1.88 (1.21–2.92)	0.005
ln C-reactive protein (per log unit)	n.s.		1.21 (1.01–1.46)	0.042
Biomarkers adjusted for basic factors				
ln Copeptin (per log unit)	1.72 (1.21–2.45)	0.003	1.61 (1.25–2.09)	<0.001
ln NT-proBNP (per log unit)	1.12 (0.88–1.42)	0.373	1.62 (1.27–2.06)	<0.001
ln hs-TnT (per log unit)	1.36 (0.88–2.11)	0.164	1.32 (1.03–1.69)	0.027
Biomarkers adjusted for basic factors and each other				
ln Copeptin (per log unit)	1.79 (1.20–2.66)	0.004	1.30 (0.96–1.76)	0.091
ln NT-proBNP (per log unit)	0.94 (0.71–1.24)	0.668	1.43 (1.07–1.89)	0.014

Median follow-up time period: 813 [Q1–3356–996] days

*Abbreviations*: *BMI* Body mass index, *CI* confidence interval, *COPD* chronic obstructive pulmonary disease, *hs-TnT* high sensitivity troponin T, *HR* hazard ratio, *n.s* not statistically significant, *NT-proBNP* N-terminal pro-B-type natriuretic peptide, vs. versus

### Copeptin concentrations and relation to diagnosis

At admission, a large portion of AECOPD patients (35% [29/84]) and acute HF patients (64% [91/143] had copeptin concentrations that exceeded the upper reference limit (14.9 pmol/L) reported for healthy subjects by the manufacturer of the current assay. The median copeptin concentration was significantly higher among acute HF patients compared to patients with AECOPD and other causes of dyspnea (22.2 [10.2–47.9] vs. 8.8 [5.2–19.7] and 8.3 [4.3–18.2] pmol/L), but NT-proBNP discriminated acute HF from non-HF related dyspnea more accurately than copeptin (AUC 0.85 [95% CI 0.81–0.89] vs. 0.71 [0.66–0.77], *p* < 0.0001). We did not find any significant difference in the concentration of copeptin or NT-proBNP between patients with AECOPD and patients with other causes of dyspnea not related to HF. By multivariate linear regression analysis across all groups (Additional file 1: Table S1) increased copeptin concentrations were independently associated with increased hs-TnT, NT-proBNP, Na^+^, male gender and reduced eGFR (r^2^ = 0.51). The correlation between copeptin and individual independent covariates were moderate for NT-proBNP (rho 0.60), hs-TNT (0.55), and eGFR (−0.52); and weak for male gender (0.27) and Na^+^ (0.24). While low Na^+^ levels were associated with lower levels of copeptin (Additional file 1: Figure S1), copeptin concentrations were measurable also among patients with hyponatremia (Na^+^ concentrations <137 mmol/L) in AECOPD (7.6 [2.7–16.0]) and acute HF (18.2 [6.3–52.6] pmol/L).

## Discussion

In this prospective observational study, we found copeptin to be a strong prognostic marker in both AECOPD and acute HF, while NT-proBNP predicted mortality only among acute HF patients. On the other hand, NT-proBNP concentrations on admission were superior to copeptin concentrations to separate patients with acute HF from patients with non-HF related dyspnea.

Sparse data exist concerning the prognostic value of copeptin in AECOPD. One previous study found that copeptin predicted mortality in a mixed population with lower respiratory tract infection, but the majority of patients in that study suffered from community-acquired pneumonia, and only 60 of 543 patients had AECOPD [19]. Another study found increasing levels of copeptin to be associated with poor prognosis in AECOPD when using a composite outcome of re-hospitalization and death [27]; however, composite outcomes are associated with uncertainty with respect to the association with individual components of the outcome [28]. A recent multicenter study found that copeptin measured in stable-state COPD predicted two-year mortality independently of selected pulmonary risk factors, and recommended a new risk assessment index including copeptin [29]. Finally, a newly published multicenter study of AECOPD patients did not find any association between copeptin and a short-term (30 days) composite outcome that included mortality, transfer to the intensive care unit, or a new visit to the emergency room. Notably, patients who required immediate intensive care unit monitoring and/or assisted ventilation (invasive or non-invasive) were excluded from the study and only 14 of 277 included patients died [30]. To clarify the prognostic properties of copeptin with respect to mortality in COPD in the acute setting we categorized unselected patients with acute dyspnea by established guidelines under the scrutiny of two experts working independently and analyzed the prognostic properties of copeptin in AECOPD and acute HF separately. From the results of our study we confirm that copeptin is a strong predictor of two-year mortality in AECOPD independently of other pulmonary and cardiac risk factors.

The prognostic utility of natriuretic peptides in COPD is controversial. In agreement with the results from one previous study [31] we did not find any predictive value of NT-proBNP regarding mortality in AECOPD. Nevertheless, other studies have indicated that NT-proBNP could be a useful prognostic marker in COPD [32, 33]. The conflicting results concerning the prognostic value of NT-proBNP in different COPD cohorts may relate to misclassification of diagnosis or differing prevalence of cardiac complications and comorbidities associated with NT-proBNP and mortality, such as pulmonary hypertension [34], cor pulmonale [35], and left ventricular dysfunction [36–38]. As no specific index can be used to diagnose AECOPD or acute HF [26, 39]. we stratified our patients according to the diagnosis made by an adjudication committee, which is considered to be the “gold-standard” strategy in order to avoid misclassification. In addition, our adjudication committee classified patients more uniformly than previous adjudication committees in similar studies [40, 41]. In our AECOPD cohort, only nine patients (11%) had a history of heart failure and the median LVEF was normal (60% [Q1–3 50–60]), indicating low prevalence of cardiac dysfunction. Thus, our results show that copeptin is a strong prognostic marker independent of cardiac pathology and support the theory that the prognostic value of NT-proBNP in COPD is related to cardiac complications and comorbidities.

As the ACE 2 Study was moderate in size, the negative result for NT-proBNP in AECOPD could also be explained by low statistical power. However, no trend for increasing mortality by NT-proBNP categories was observed in Kaplan-Meier survival plots (Fig. 2). We could not find a statistical significant difference between the prognostic accuracy of copeptin and NT-proBNP by ROC-AUC in AECOPD, in contrast to the results obtained by Cox proportional hazard regression analysis, but this inconsistency probably relates to inferior statistical properties when comparing predictors by ROC-AUC as opposed to regression analysis [42]. NRI analyses found copeptin, but not NT-proBNP, to reclassify a significant proportion of patients into their correct risk strata on top of a basic clinical model. This emphasizes the superior prognostic value of copeptin over NT-proBNP levels in AECOPD. Accordingly; our data support copeptin as the preferred prognostic biomarker concerning mortality in patients with AECOPD.

The prognostic accuracies of copeptin and NT-proBNP seem comparable in HF. In previous studies of chronic HF, copeptin has been associated with short- and long-term prognostic outcomes independently of other risk factors including natriuretic peptides, and in some of these studies copeptin also predicted the outcome with higher accuracy than BNP and NT-proBNP [16–18]. In our study, we found copeptin to be a strong predictor of mortality in acute HF patients independently of basic risk factors, but after adjustment for NT-proBNP the prognostic effect of copeptin by Cox regression was not significant (Table 2). In contrast, we did not find any significant difference between the prognostic accuracy of copeptin and NT-proBNP estimated by ROC-AUC or improvement in the overall predictive accuracy when NT-proBNP was added to the multivariate Cox regression model already including copeptin. The most likely explanation for these findings is that copeptin and NT-proBNP carry much of the same prognostic information in acute HF. Hence, copeptin also appears to be a valid alternative to NT-proBNP with respect to mortality risk evaluation in acute HF.

NT-proBNP concentrations were superior to copeptin concentrations for the discrimination of acute HF from other causes of dyspnea in our patients. This is not surprising from a theoretical viewpoint as NT-proBNP is released mainly as a result of myocardial stretch [43], while copeptin release most likely is stimulated by the same mechanisms known to stimulate AVP secretion that are less specific for heart failure [6]. The finding that NT-proBNP is a better diagnostic marker of acute HF than copeptin is in line with our main finding that copeptin is a better prognostic marker than NT-proBNP in AECOPD patients.

The mechanisms responsible for increased copeptin release in AECOPD are unclear. The median concentration of copeptin found in AECOPD (8.8 [5.2–19.7]) is higher than what has been reported in healthy subjects recruited from the general population (3.7–4.2 pmol/L) [12, 44]. A functioning osmotic regulation of copeptin release is indicated by the positive correlation between copeptin and Na^+^ concentrations in our patients (Additional file 1: Figure S1), but contrary to what is expected under normal osmotic regulation [45], we found that copeptin is not clearly suppressed among AECOPD or acute HF patients with Na^+^ concentrations <137 mmol/L (7.6 [2.7–16.0] and 18.2 [6.3–52.6] pmol/L, respectively). The missing suppression of copeptin among hyponatremic patients suggest the presence of an non-osmotic stimulation of copeptin and AVP release that could also explain the high prevalence of hyponatremia in AECOPD (27%) and acute HF (20%) previously documented in the ACE 2 Study [23]. In HF patients, arterial under-filling is a strong stimulant of non-osmotic copeptin and AVP release via baroreceptors [7], but this pathway seem less likely in AECOPD patients with close to normal cardiac function. Another relevant question is whether the concentration of copeptin increases during AECOPD compared to stable-state COPD. Interestingly, one previous study of COPD patients could not find any difference between copeptin levels in stable-state and exacerbations [29]. We were, however, not able to explore this question further as stable-state copeptin measures were not included in the ACE 2 study design. Clearly, further studies are needed to explore the mechanisms that stimulate copeptin and AVP secretion in COPD.

## Conclusion

Copeptin is a strong prognostic marker in patients with acute exacerbation of chronic obstructive pulmonary disease (AECOPD) and acute heart failure (HF), while NT-proBNP concentrations predict mortality only among patients with acute HF. Accordingly, copeptin could be preferable to NT-proBNP for risk stratification in AECOPD and mixed populations that include both AECOPD and acute HF patients. Copeptin concentrations are significantly higher in patients with acute HF compared to other etiologies of acute dyspnea, but NT-proBNP is superior to copeptin for diagnosing HF in the acute setting.

## Additional file

Additional file 1: Table S1. Linear regression for log (ln) copeptin (*n* = 314). **Table S2.** Univariate proportional Cox regression analysis. **Table S3.** Multivariate proportional Cox regression analysis for the non-HF, non-AECOPD group. **Figure S1.** Correlation between copeptin and Na^+^. (DOCX 267 kb)

## Abbreviations

ACE Study: Akershus Cardiac Examination Study
AECOPD: Acute exacerbation of chronic obstructive pulmonary disease
CI: Confidence interval
CKD-EPI: Chronic Kidney Disease Epidemiology Collaboration
COPD: Chronic obstructive pulmonary disease
CRP: C-reactive protein
eGFR: estimated glomerular filtration rate
GOLD: Global initiative for Chronic Obstructive Lung Disease
HF: Heart failure
HR: Hazard ratio
hs-TnT: High-sensitivity troponin T
LVEF: Left ventricular ejection fraction
NRI: Net reclassification index
NT-proBNP: N-terminal pro-hormone of B-type natriuretic peptide
Q: Quartile
ROC-AUC: Area under receiver operating curves
SD: Standard deviation
Vs: versus
