# Supplementary material for: Prognostic and diagnostic significance of copeptin in acute exacerbation of chronic obstructive pulmonary disease and acute heart failure: data from the ACE 2 study
Source: Respir Res. 2017 Nov 3;18:184. doi: 10.1186/s12931-017-0665-z (PMC5670515; doi:10.1186/s12931-017-0665-z)

Supplementary data to

Prognostic and diagnostic significance of copeptin in acute dyspnea: data from the Akershus Cardiac Examination (ACE) 2 Study

Jacob A. Winther MD^1,2*^, Jon Brynildsen MD^1,2^, Arne Didrik Høiseth MD, PhD^1,2^, Heidi Strand MSc^4^, Ivar Følling MD^1,2^, Geir Christensen MD, PhD, MHA^3^, Ståle Nygård PhD^5^, Helge Røsjø MD, PhD^1,2^, Torbjørn Omland MD, PhD, MPH^1,2^

^1^ Division of Medicine, Akershus University Hospital, Lørenskog, Norway

^2^ Institute of Clinical Medicine, University of Oslo, Oslo, Norway

^3^ Institute for Experimental Medical Research, Oslo University Hospital and University of Oslo, Oslo, Norway

^4^ Division of Diagnostics and Technology, Akershus University Hospital, Lørenskog, Norway

^5^ Bioinformatics Core Facility, Institute for Medical Informatics, Oslo University Hospital and University of Oslo, Oslo, Norway

# Supplementary Table S1. Linear regression for log (ln) copeptin (n=314)

|  | Coefficient (95% CI) | P |
| --- | --- | --- |
| Age (years) | 0.03 (0.03-0.04) | <0.001 |
| Male sex | 0.54 (0.30 -0.77) | <0.001 |
| BMI (kg/m^2^) | -0.01 (-0.03-0.01) | 0.193 |
| Heart rate (per 5 beats/minute) | -0.01 (-0.04-0.01) | 0.340 |
| Mean arterial pressure (per 5 mmHg) | -0.01 (-0.05-0.02) | 0.562 |
| Peripheral edema | 0.71 (0.48-0.95) | <0.001 |
| PaO_2_ (kPa) ^†^ | -0.03 (-0.08-0.03) | 0.354 |
| NYHA class IV vs. II-III | 0.39 (0.15-0.64) | 0.001 |
| LVEF (%) ^†^ | -0.03 (-0.04--0.02) | <0.001 |
| FEV_1_ % of predicted (per 10%) ^‡^ | 0.02 (-0.11-0.14) | 0.709 |
| FEV_1_/FVC (per 10%) ^‡^ | 0.10 (-0.04-0.24) | 0.139 |
| Current smoker | -0.43 (-0.70--0.16) | 0.002 |
| Diabetes mellitus | 0.52 (0.23-0.81) | 0.001 |
| Chronic heart failure | 0.98 (0.74-1.22) | <0.001 |
| Coronary artery disease | 0.62 (0.38-0.87) | <0.001 |
| Hypertension | 0.36 (0.11-0.61) | 0.005 |
| COPD | -0.12 (-0.37-0.12) | 0.317 |
| Beta-blocker | 0.71 (0.48-0.94) | <0.001 |
| ACEi/ARB | 0.58 (0.35-0.82) | <0.001 |
| Diuretic therapy | 0.90 (0.68-1.12) | <0.001 |
| Glucose (mmol/L) | 0.06 (0.02-0.09) | 0.002 |
| K^+^ (mmol/L) | 0.37 (0.15-0.59) | 0.001 |
| Na^+^ (mmol/L) ^*^ | 0.06 (0.03-0.09) | <0.001 |
| eGFR (mL/min) | -0.02 (-0.02--0.01) | <0.001 |
| ln C-reactive protein (per log unit) | 0.07 (0.00-0.14) | 0.044 |
| ln hs-TnT (per log unit) | 0.52 (0.44-0.61) | <0.001 |
| ln NT-proBNP (per log unit) | 0.35 (0.30-0.40) | <0.001 |
| Acute HF | 0.83 (0.61-1.06) | <0.001 |
| AECOPD | -0.50 (-0.76--0.23 | <0.001 |
| Multivariate analysis |  |  |
| Male sex | 0.27 (0.06-0.49) | 0.011 |
| eGFR (mL/min) | -0.08 (-0.01--0.00) | <0.001 |
| Na^+^ (mmol/L) ^*^ | 0.04 (0.02-0.06) | 0.001 |
| ln hs-TnT (per log unit) | 0.19 (0.08-0.31) | 0.001 |
| ln NT-proBNP (per log unit) | 0.21 (0.13-0.28) | <0.001 |

^†^ Missing data > 10 %); ^‡^ Missing data > 50 %; ^*^ Corrected for hyperglycemia by the Hillier formula.

Abbreviations: ACEi, angiotensin-converting-enzyme inhibitor; ARB, angiotensin II receptor blocker; BMI, Body mass index; CI, confidence interval; COPD, chronic obstructive pulmonary disease; FEV1, forced expiratory volume in one second; FVC, forced vital capacity; eGFR, estimated glomerular filtration rate (CKD-EPI); hs-TnT, high sensitivity troponin T; LVEF, left ventricular ejection fraction; NT-proBNP, N-terminal pro-B-type natriuretic peptide; NYHA, New York Heart Association; P_a_O_2_, partial pressure of oxygen; vs., versus.

# Supplementary Table S2. Univariate proportional Cox regression analysis

|  | Acute exacerbation of COPD (n=84) | | Acute HF  (n=143) | | Non-HF, non-AECOPD  (n=87) | |
| --- | --- | --- | --- | --- | --- | --- |
|  | HR (95% CI)^*^ | P | HR (95% CI)^*^ | P | HR (95% CI)^*^ | P |
| Age (years) | 1.03 (0.99-1.07) | 0.100 | 1.04 (1.02-1.07) | 0.002 | 1.07 (1.02-1.12) | 0.003 |
| Male sex | 1.92 (0.99 -3.75) | 0.056 | 0.53 (0.32-0.86) | 0.010 | 1.12 (0.37-3.34) | 0.844 |
| BMI (kg/m^2^) | 0.90 (0.84-0.96) | 0.002 | 0.94 (0.89-0.99) | 0.012 | 0.94 (0.87-1.03) | 0.176 |
| Heart rate (per 5 beats/minute) | 1.05 (0.96-1.14) | 0.315 | 0.98 (0.94-1.03) | 0.401 | 0.96 (0.84-1.09) | 0.516 |
| Mean arterial pressure (per 5 mmHg) | 0.93 (0.83-1.04) | 0.191 | 0.89 (0.83-0.95) | 0.001 | 0.92 (0.76-1.11) | 0.363 |
| Peripheral edema | 1.15 (0.58-2.26) | 0.691 | 1.33 (0.81-2.17) | 0.257 | 2.29 (0.70-7.50) | 0.172 |
| NYHA class IV vs. II- III | 1.11 (0.56-2.19) | 0.772 | 2.01 (1.23-3.29) | 0.006 | 1.33 (0.41-4.32) | 0.639 |
| LVEF (%) | 0.97 (0.93-1.02) ^†^ | 0.264 | 1.00 (0.98-1.02) | 0.872 | 0.94 (0.87-1.01) ^†^ | 0.083 |
| FEV_1_ % of predicted (per 10%) | 0.78 (0.61-0.99) | 0.038 | n.a. ^‡^ |  | n.a. ^‡^ |  |
| FEV_1_/FVC (per 10%) | 0.74 (0.58-0.96) ^†^ | 0.026 | n.a. ^‡^ |  | n.a. ^‡^ |  |
| Current smoker | 1.74 (0.88-3.47) | 0.114 | 0.88 (0.48-1.62) | 0.681 | 0.42 (0.09-1.92) | 0.265 |
| Diabetes mellitus | 0.46 (0.11-1.93) | 0.289 | 1.78 (1.08-2.95) | 0.024 | 1.78 (1.08-2.95) | 0.024 |
| Chronic heart failure | 2.21 (0.85-5.74) | 0.104 | 1.43 (0.86-2.38) | 0.167 | 1.40 (0.18-10.85) | 0.747 |
| Coronary artery disease | 0.91 (0.43-1.95) | 0.817 | 1.05 (0.65-1.70) | 0.854 | 0.62 (0.08-4.74) | 0.641 |
| Hypertension | 1.08 (0.53-2.20) | 0.843 | 0.83 (0.51-1.36) | 0.465 | 3.56 (1.18-10.70) | 0.024 |
| COPD | n.a. |  | 1.85 (1.14-3.01) | 0.013 | 4.13 (1.27-13.44) | 0.083 |
| Beta-blocker | 1.48 (0.76-2.90) | 0.250 | 1.28 (0.77-2.13) | 0.337 | 3.38 (1.14-10.08) | 0.029 |
| ACEi/ARB | 1.39 (0.70-2.76) | 0.349 | 1.52 (0.90-2.55) | 0.119 | 3.42 (1.15-10.21) | 0.027 |
| Diuretic therapy | 1.04 (0.36-2.95) | 0.948 | 0.59 (0.25-1.36) | 0.214 | 1.309 (0.40-4.24) | 0.659 |
| K^+^ (mmol/L) | 2.68 (1.24-5.82) | 0.013 | 2.09 (1.39-3.14) | <0.001 | 4.79 (1.75-13.10) | 0.002 |
| Na^+^ (mmol/L) | 0.99 (0.93-1.05) | 0.743 | 0.98 (0.93-1.03) | 0.365 | 0.95 (0.85-1.06) | 0.328 |
| eGFR (mL/min) | 1.00 (0.98-1.02) | 0.811 | 0.98 (0.97-0.99) | <0.001 | 1.01 (1.00-1.03) | 0.061 |
| ln C-reactive protein (per log unit) | 1.13 (0.94-1.38) | 0.197 | 1.26 (1.06-1.50) | 0.010 | 0.98 (0.76-1.27) | 0.879 |
| ln hs-TnT (per log unit) | 1.40 (0.92-2.12) | 0.114 | 1.37 (1.10-1.71) | 0.005 | 2.11 (1.32-3.36) | 0.002 |
| ln NT-proBNP (per log unit) | 1.07 (0.85-1.35) | 0.564 | 1.53 (1.24-1.89) | <0.001 | 1.53 (1.12-2.09) | 0.008 |
| ln Copeptin (per log unit) | 1.79 (1.25-2.57) | 0.002 | 1.64 (1.29-2.09) | <0.001 | 2.63 (1.44-4.80) | 0.002 |

^*^HR is expressed per unit unless otherwise specified (yes vs. no for binary variables); ^†^ Missing data (10-35%); ^‡^ Missing data > 85%.

Abbreviations: ACEi, angiotensin-converting-enzyme inhibitor; ARB, angiotensin II receptor blocker; BMI, Body mass index; CI, confidence interval; COPD, chronic obstructive pulmonary disease; eGFR, estimated glomerular filtration rate (CKD-EPI); FEV1, forced expiratory volume in one second; FVC, forced vital capacity; HF, heart failure; hs-TnT, high sensitivity troponin T; HR, hazard ratio; LVEF, left ventricular ejection fraction; n.a., not applicable; NT-proBNP, N-terminal pro-B-type natriuretic peptide; NYHA, New York Heart Association; vs., versus.

# Supplementary Table S3. Multivariate proportional Cox regression analysis for the non-HF, non-COPD group

|  | **Non-HF, non-COPD**  **(n=87)** | |
| --- | --- | --- |
|  | **HR (95% CI)** | **P** |
| **Basic risk factors** |  |  |
| Age (per year) | 1.06 (1.01-1.11) | 0.016 |
| K^+^ (per mmol/l) | 2.95 (1.01-8.56) | 0.047 |
| **Biomarkers adjusted for basic factors** |  |  |
| ln Copeptin (per log unit) | 1.74 (0.85-3.56) | 0.132 |
| ln NT-proBNP (per log unit) | 1.06 (0.71-1.58) | 0.784 |
| ln hs-TnT (per log unit) | 1.43 (0.76-2.66) | 0.265 |

# Supplementary Figure S1. Correlation between copeptin and Na^+^


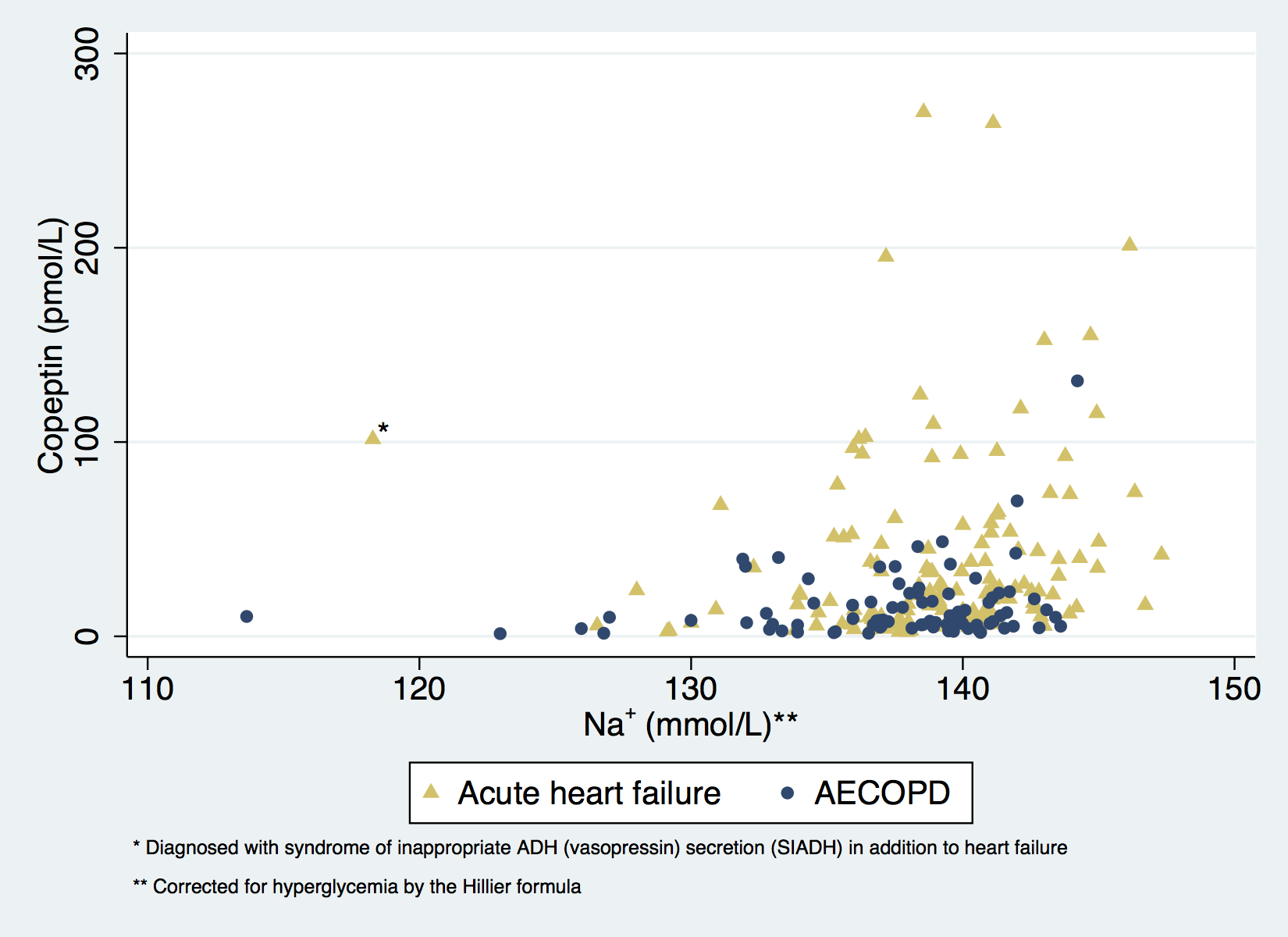

Supplement: Additional file 1: Table S1. — Linear regression for log (ln) copeptin (n = 314). Table S2. Univariate proportional Cox regression analysis. Table S3. Multivariate proportional Cox regression analysis for the non-HF, non-AECOPD group. Figure S1. Correlation between copeptin and Na+. (DOCX 267 kb) [file 12931_2017_665_MOESM1_ESM.docx]
